# Culture-enriched human gut microbiomes reveal core and accessory resistance genes

**DOI:** 10.1186/s40168-019-0669-7

**Published:** 2019-04-05

**Authors:** Frédéric Raymond, Maurice Boissinot, Amin Ahmed Ouameur, Maxime Déraspe, Pier-Luc Plante, Sewagnouin Rogia Kpanou, Ève Bérubé, Ann Huletsky, Paul H. Roy, Marc Ouellette, Michel G. Bergeron, Jacques Corbeil

**Affiliations:** 1 École de nutrition, Faculté des sciences de l’agriculture et de l’alimentation, Université Laval, Québec City, Canada; 2 Institut sur la nutrition et les aliments fonctionnels, Québec, Canada; 3 Centre de Recherche en Infectiologie de l’Université Laval, Axe Maladies Infectieuses et Immunitaires, Centre de Recherche du CHU de Québec-Université Laval, Québec City, Canada; 4 Département de Microbiologie, Infectiologie et d’Immunologie, Faculté de Médecine, Université Laval, Québec City, Canada; 5 Centre de recherche en données massives, Université Laval, Québec City, Canada; 6 Département de médecine moléculaire, Faculté de Médecine, Université Laval, Québec City, Canada; 7 Département de biochimie, de microbiologie et de bio-informatique, Faculté des Sciences et de Génie, Université Laval, Québec City, Canada

**Keywords:** Microbiome, Antibiotic resistance, Pangenome, Metagenomics assembly comparative genomics

## Abstract

**Background:**

Low-abundance microorganisms of the gut microbiome are often referred to as a reservoir for antibiotic resistance genes. Unfortunately, these less-abundant bacteria can be overlooked by deep shotgun sequencing. In addition, it is a challenge to associate the presence of resistance genes with their risk of acquisition by pathogens. In this study, we used liquid culture enrichment of stools to assemble the genome of lower-abundance bacteria from fecal samples. We then investigated the gene content recovered from these culture-enriched and culture-independent metagenomes in relation with their taxonomic origin, specifically antibiotic resistance genes. We finally used a pangenome approach to associate resistance genes with the core or accessory genome of *Enterobacteriaceae* and inferred their propensity to horizontal gene transfer*.*

**Results:**

Using culture-enrichment approaches with stools allowed assembly of 187 bacterial species with an assembly size greater than 1 million nucleotides. Of these, 67 were found only in culture-enriched conditions, and 22 only in culture-independent microbiomes. These assembled metagenomes allowed the evaluation of the gene content of specific subcommunities of the gut microbiome. We observed that differentially distributed metabolic enzymes were associated with specific culture conditions and, for the most part, with specific taxa. Gene content differences between microbiomes, for example, antibiotic resistance, were for the most part not associated with metabolic enzymes, but with other functions. We used a pangenome approach to determine if the resistance genes found in *Enterobacteriaceae,* specifically *E. cloacae* or *E. coli*, were part of the core genome or of the accessory genome of this species. In our healthy volunteer cohort, we found that *E. cloacae* contigs harbored resistance genes that were part of the core genome of the species, while *E. coli* had a large accessory resistome proximal to mobile elements.

**Conclusion:**

Liquid culture of stools contributed to an improved functional and comparative genomics study of less-abundant gut bacteria, specifically those associated with antibiotic resistance. Defining whether a gene is part of the core genome of a species helped in interpreting the genomes recovered from culture-independent or culture-enriched microbiomes.

**Electronic supplementary material:**

The online version of this article (10.1186/s40168-019-0669-7) contains supplementary material, which is available to authorized users.

## Background

The widespread use of antibiotics has been accompanied by the appearance and dissemination of antibiotic-resistant bacteria, which is an alarming public health problem [1, 2]. Moreover, antibiotics not only affect the pathogenic bacteria but also have profound effects on commensal bacteria involved in human health [3]⁠. It has been proposed that the human gut microbiota serves as a reservoir of antibiotic-resistance genes (resistome) [4]. Genomic approaches substantially increased our knowledge about short- and long-term impact of antibiotics on the composition and functions of human gut microbiota [5–8]. Antibiotics can affect low-abundance taxa [9]⁠, thus requiring increased sequencing efforts for proper genome assembly and identification of strain and functions [10, 11].

While researchers routinely rely on culture-independent sequencing approaches to study the microbiome [12]⁠, there is a need to include cultivation techniques, combined with genome sequencing, to uncover less-abundant microorganisms, including bacteria, archaea, and fungi [13–15]. Culture on different media in Petri dishes, followed by colony picking, allowed the discovery and sequencing of the genome of hundreds of new species [16, 17]. This culture-based approach can potentially allow the characterization of bacteria that are less abundant in the microbiota but still play a significant role in maintaining health [18]. Similarly, Lau and collaborators have grown microbiota on 33 solid media, both under aerobic and anaerobic conditions, to capture the diversity of the microbiome through culture [19]. Others have grown bacteria on solid or in liquid culture media to complement microbiome sequencing and to find culture conditions that allow the obtention of an accurate community representation of the complete microbiota [20, 21].

In this work, we set out to go further in the use of microbial culture as a complementary tool for microbiome analysis. We cultured stool samples in liquid media to complement information from culture-independent microbiome (CIM) sequencing, thus allowing deep shotgun sequencing of culture-enriched microbiome (CEM). CEM permitted metagenome assembly of selected subcommunities from the gut microbiome. We chose the MCDA broth [22] as a base medium under two atmospheric conditions (aerobic with 5% CO2 and anaerobic), and in the presence or absence of the antibiotic cefoxitin, a second-generation cephalosporin. We investigated how these different culture conditions allowed the metagenomic assembly of a greater segment of the gut microbiome. Deeper insight on the gut resistome was obtained from the CEM, especially when using a pangenome perspective to interpret the antibiotic resistance genes found in *Enterobacteriaceae.*

## Methods

### Ethics statement and sampling

The study protocol was approved by the ethical committee of the CHU de Québec–Université Laval. Informed written consent was obtained from participants. Healthy volunteer stool samples used in this study have been described in previous work [9].

### Study design

The samples used in this study were collected from healthy participants that received oral doses of the antibiotic cefprozil for 7 days as described [9]. All samples were brought to the laboratory within 2 h of collection and placed immediately in an anaerobic chamber for processing. Samples were collected before antibiotic treatment (day 0), at the end of the treatment (day 7), and 3 months later (day 90). Culture-independent microbiome (CIM) sequencing was performed on these stool samples. Description of the collection, DNA extraction, and sequencing of CIM was described by Raymond and collaborators [9]. The same stool samples were used for culture-enriched microbiome (CEM) sequencing as described in the next section.

### Cultivation and DNA extraction

Stool samples from 24 healthy volunteers, harvested at day 0 and day 7 were used for the generation of CEM. The fecal material (1 g) was suspended in 15 ml PBS containing 0.1% cysteine (PBSc) and supernatant was obtained as previously described [23]. The supernatant was used to inoculate MCDA broth, which is based on brain-heart infusion (BHI) broth supplemented with haemin, vitamin K, L-cystine, lactate, and pyruvate [22]. Four CEM conditions for each sample were used in this study: (i) maximum enrichment broth anaerobic culture (MEB-ANA), (ii) maximum enrichment broth aerobic culture (air enriched with 5% CO_2_) (MEB-CO2), (iii) anaerobic culture in the presence of 32 μg/ml cefoxitin (FOX-ANA), and (iv) aerobic (with 5% CO_2_) culture in the presence of 32 μg/ml cefoxitin (FOX-CO2). Anaerobic cultures were incubated in a Bactron anaerobic chamber (Sheldon Manufacturing, Inc., Cornelius, Oregon, USA). Cultures were incubated at 35 °C for 7 days to favor the growth of bacteria with longer generation time [23]. All cultures were conserved at − 80°C in the presence of 15% glycerol.

Crude DNA extracts were obtained from 1.5 ml of frozen cultures using the BD GeneOhm™ Lysis Kit as recommended by the manufacturer (BD Diagnostics-GeneOhm, Québec, Canada). Genomic DNA was purified using the Biosprint^TM^ 15 DNA Blood Kit (Qiagen, Mississauga, Ontario, Canada) on a KingFisher® ML instrument according to the manufacturer’s instructions (Thermo Fisher Scientific, Waltham, MA, USA). The quantity and quality of isolated DNA were measured using a NanoDrop ND-1000 spectrophotometer (Thermo Fisher Scientific, Waltham, MA, USA), agarose gel electrophoresis, and a QuantiFluor® dsDNA System (Promega). Extraction of CIM also included a bead-beating step and was performed with MO BIO PowerMax Soil DNA Extraction Kit (MO BIO Laboratories, Carlsbad, CA, USA) using a modified protocol [9].

### Shotgun sequencing

For each sample, 50 ng of purified genomic DNA were used to construct a sequencing library using a Nextera DNA sample preparation kit (Illumina, San Diego, CA, USA). Library validation was performed using Agilent Bioanalyzer 2100 high sensitivity DNA chips (Agilent Technologies, Santa Clara, CA, USA). Eight libraries with different indexes were pooled in equimolar concentration (2 nM) prior to cluster generation in a cBot system (Illumina). Each pooled sample was sequenced in one lane using an Illumina HiSeq 1000 high-throughput sequencer with 2 × 101 bp paired-end sequencing according to the manufacturer’s instructions. A total of 24 lanes were sequenced for 192 libraries. An average of 3.2 Gb was sequenced per CEM culture condition for each sample, for a total of 611 Gb. Sequences of culture-enriched microbiomes (CEM) are available in European Nucleotide Archive PRJEB28237. Sequencing and assembly statistics of CEM are shown in Additional file 1: Table S1. We also compared CEM sequences to culture-independent microbiome (CIM) sequences obtained from the same participants in a previous study [9, 11]. An average of 12.6 Gb were sequenced per CIM sample for a total of 907 Gb. Sequences of CIM are available in European Nucleotide Archive PRJEB8094.

### Bioinformatics

All genomic paired-end reads were assembled and profiled for taxonomy and resistance genes using the RAY Meta 2.2.1 assembler [24, 25] with the same protocol and reference database as in previous work [9]. Statistical analysis was done with R 3.0.2. Gene finding, resistance gene annotation, and contig identification were performed as described in supplementary materials of Raymond et al. [9]. In summary, putative genes were aligned to the MERGEM antibiotic resistance gene database and were annotated as resistance genes when similarity was more than 70% of the amino acid sequence.

Diversity indices were computed using the Vegan R package. EC number annotation was performed by comparing putative proteins to the Brenda database using CD-hit-2D with a 95% identity threshold [26, 27]. Scripts for EC annotation are available on GitHub (https://github.com/fredericraymond/GeneAnnotationTools). Comparison of gene content was performed for putative protein sequences at a threshold of 85% identity using CD-HIT V4.7 [26].

For pangenome analysis, genome collections for selected species were downloaded from NCBI. Genomes were annotated for resistance genes using the same protocol as for CIM and CEM, using the MERGEM database as reference [9]. Comparison of CIM and CEM are based on the presence/absence of specific antibiotic resistance genes.

## Results

### Culture of fecal samples allows the sequencing of less-abundant bacteria

**Fig. 1:**
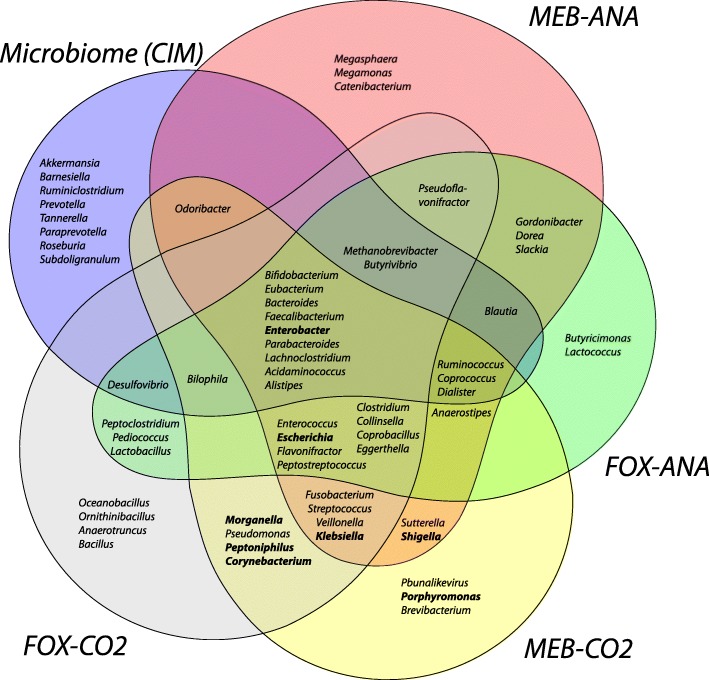
Comparison of microbial genera observed in the four culture-enriched conditions and in the culture-independent microbiome metagenomes. Genera shown were at least 1% of the microbial community in at least one sample of the five conditions that were sequenced. Genera in bold were identified as mucosally associated by Albenberg and collaborators [28]

Stool samples were grown for 7 days under four culture conditions using the same base media (MCDA broth) and were sequenced using shotgun metagenomics to generate CEM. Previously published culture-independent microbiome (CIM) sequencing from the same stool samples was used for comparison [9]. Taxonomic profiling shows that CEM sequencing gives a different portrait of communities compared to CIM (Fig. 1, Additional file 2: Figure S1 and Additional file 3: Table S2). We used the Shannon diversity index to compare the overall distribution of species observed in CIM and in CEM under the four culture conditions (Additional file 2: Figure S1A). Culture in the MEB-ANA and MEB-CO_2_ conditions had higher diversity than CIM, FOX-ANA, or FOX-CO_2_ conditions (*p* = 3.7e-11, *t* test). Culture in MEB conditions of samples from participants not exposed to antibiotics had significantly higher diversity than culture samples from participants exposed to cefprozil (*p* = 2.4e-6, *t* test), which was also the case for CIM (*p* = 0.004, *t* test). This difference was not observed when culture conditions included antibiotics (*p* = 0.98, *t* test).

Of the 64 genera that were globally observed, 38 (59% of all genera) were found at more than 1% of the community in culture only (Fig. 1). Indeed, the relative abundance of *Escherichia* is 100 to 1000 times higher in MEB-CO_2_ culture than in CIM. *Enterococcus* and *Peptostreptococcus* were detected in negligible amounts in CIM while they represented up to 1–10% in CEM. Difficult to culture *Methanobrevibacter* grew up to 5% in CO_2_ conditions. By contrast, *Akkermansia*, *Alistipes*, and *Prevotella* were 10–100 times more abundant in the CIM than under the CEM culture conditions. Interestingly, several genera observed in culture (Fig. 1, in bold), especially under the MEB-CO_2_ condition, were previously found to be associated with the mucus layer of the intestine [28].

Assembled metagenomes were annotated based on their k-mer similarity to known genomes thus enabling identification of their taxonomic provenance. The total assembly size of each species was calculated for all samples and conditions (Additional file 4: Table S3 and Additional file 5: Figure S2). Overall, 187 species had an assembly size greater than 1 Mbp for at least one condition and 22 species reached it only in CIM and 67 only in CEM. A total of 148 species had an assembly size greater than 2 Mbps (22 only in CIM and 48 only in CEM), 101 greater than 3 Mbps (29 only in CIM and 33 only in CEM), and 58 greater than 4 Mbps (15 only in CIM and 17 only in CEM). In all cases, more species were assembled above these thresholds in CEM than in CIM. Using culture approaches with stools allowed to sequence the genome of a greater number of species not sequenced originally. We could then perform a better evaluation of the gene content of taxa that are less abundant in the microbiome but grow well in selected culture conditions. The most notable example is the *Enterobacteriaceae* like *Escherichia*, which are well known antibiotic resistance gene carriers and likely have a significant impact on the global gut resistome*.*

### Gene content of culture-enriched microbiota

**Fig. 2:**
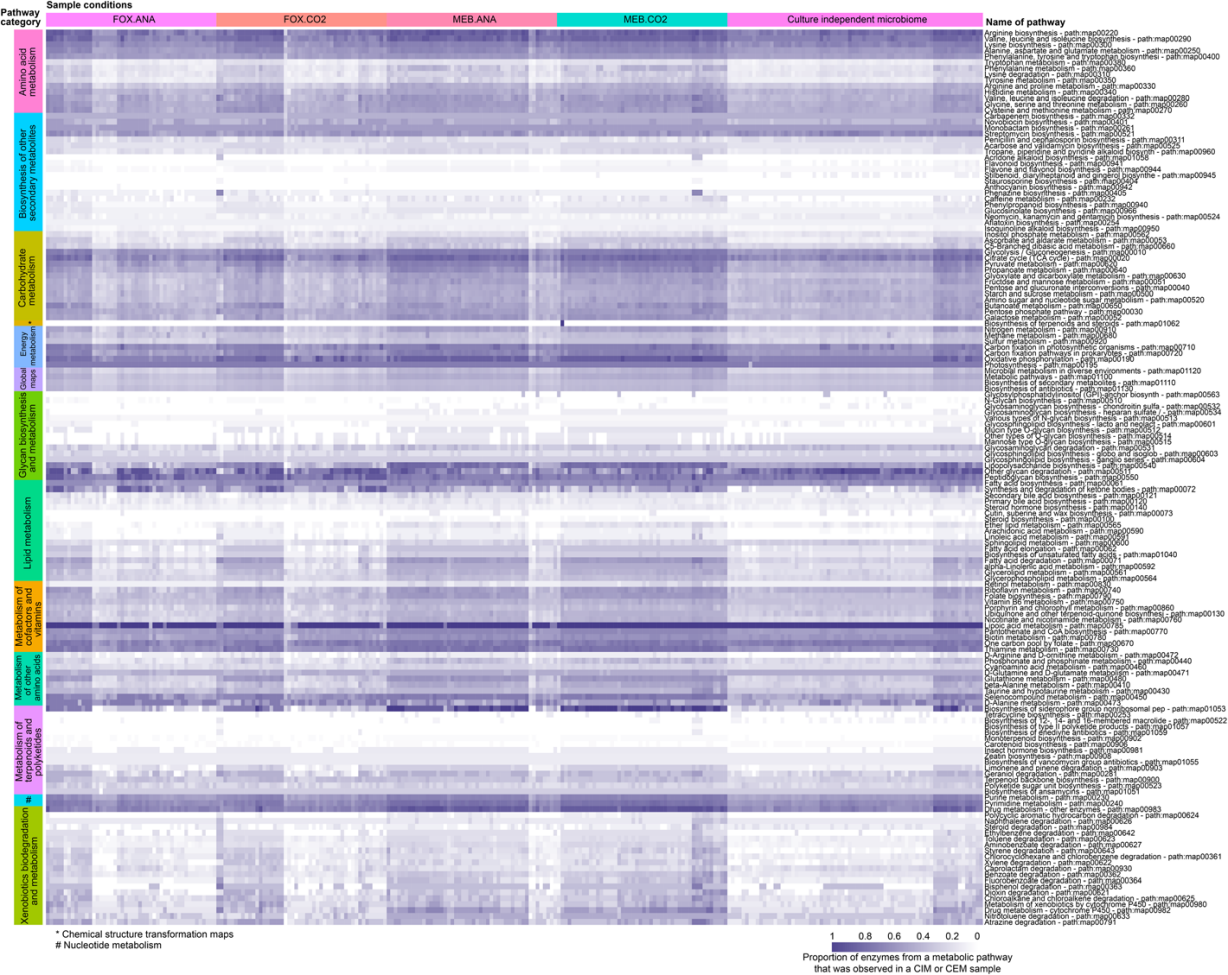
Coverage of metabolic pathways in culture-independent and culture-enriched metagenomes. Coverage is the proportion of enzymes from a metabolic pathway that was observed in a sample. Metabolic pathways are hierarchically clustered within pathway categories, which are indicated by a color code. Columns of the heatmap are samples hierarchically clustered within CIM and CEM conditions

We investigated how culture conditions affected the metabolic pathways observed in the CEM compared to CIM. Assembled CIM and CEM were thus annotated with genes associated with metabolic pathways, based on Enzyme Commission (EC) annotation (Fig. 2). We detected 1856 different enzymes (different EC numbers) in CIM samples while 2176 different enzymes were annotated in the CEM samples. Overall, 1828 enzymes were found both in CIM and in CEM. Although the sequencing depth and assembly size of CEM were respectively 4.2 and 2.5 times lower than CIM, 348 enzymes were unique to CEM and only 28 were specific to CIM. These enzymes belonged to a total of 170 metabolic pathways, 151 of which were found under all culture conditions and in CIM, in at least one sample (Additional file 6: Figure S3). Pathways not observed in all conditions were associated with the biosynthesis of secondary metabolites.

**Fig. 3:**
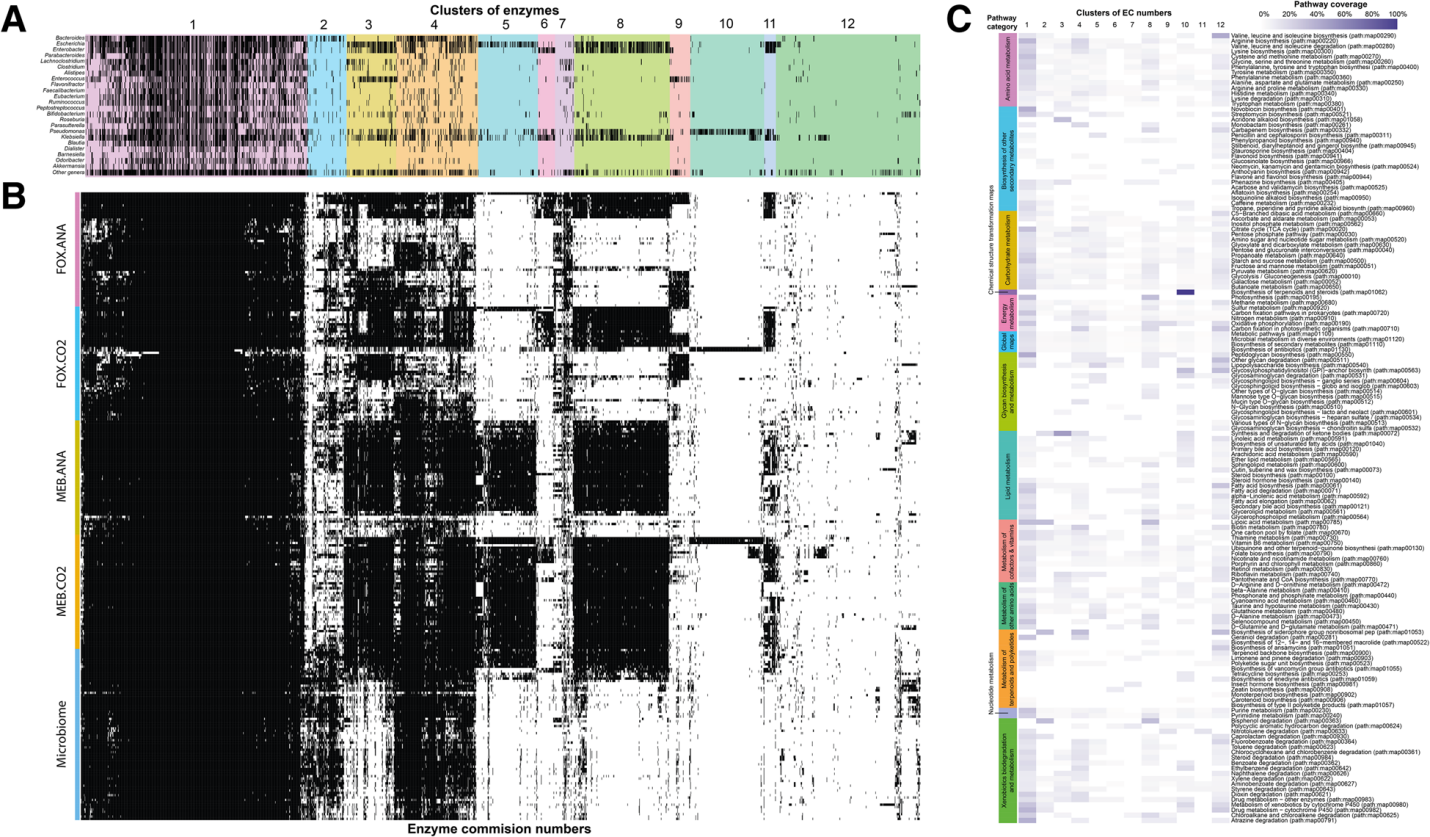
Distribution of enzymes involved in metabolic pathways in culture-independent and culture-enriched metagenomes. Enzymes were annotated in the metagenomic assemblies of CIM and CEM samples based on Enzyme Commission groups, and their taxonomical origin was inferred based on contig sequences. **a** The distribution of the taxonomical origin of enzymes at the genus rank. **b** The distribution of enzymes in the CIM and CEM samples. In both cases, black bars indicate the presence of an enzyme-associated genus or sample. Panels A and B are meant to be interpreted together as they share the same horizontal axis, which represents individual enzyme groups. Colors in **a** are clusters of EC numbers based on their presence or absence in CIM and CEM. **c** Coverage of pathways in the 12 clusters defined in **a** and **b**. The coverage represents the percentage of genes from the pathway that was observed in a cluster. Colored bars on the left of **c** are pathway categories

Enzymes were clustered based on their distribution in the CIM and CEM using k-means clustering. Each enzyme was also annotated with the taxonomic origin of the contigs on which it was found (Fig. 3a). The putative taxonomic origin of enzymes was coherent with clustering enzymes into 12 groups, and this clustering reflected the selection of taxa by culture (Fig. 3a, b). The proportion of samples positive for enzymes per conditions and clusters is shown in Additional file 7: Figure S4. In addition, metabolic pathway coverage was calculated for the 12 clusters (Fig. 3c). The most ubiquitous enzymes belonged to cluster 1, from which 587 enzymes were shared by more than 81.4% of samples, both in CIM and CEM. These enzymes could be associated with approximately 230 genera. Pathway categories with lesser representation in cluster 1 were biosynthesis of secondary metabolites, glycan biosynthesis and metabolism, and metabolism of terpenoids and polyketides. Pathways absent from cluster 1 were most of the time represented in other clusters, suggesting that they would be taxa-specific. Although they were also observed in some CEM samples, enzymes from clusters 2 and 4 were strongly represented in CIM. The most frequent taxonomic origin of enzymes found in cluster 2 was *Bacteroides* while enzymes from Cluster 4 were observed in both *Bacteroides* and *Enterobacteriaceae.* Cluster 9 was associated with *Enterococcus,* which was mostly observed in CEM in the presence of cefoxitin. Interestingly, in cluster 10, three cultures under CO_2_-enriched atmosphere from the same participant, in the presence or absence of cefoxitin, yielded almost pure cultures of *Pseudomonas aeruginosa.* Cluster number 12 was not associated with any specific condition or taxa and included genes that were either rare in CIM or present in less-abundant bacterial species, which are difficult toassemble. Overall, these functional analyses suggest that metabolic pathways are strongly correlated with the taxonomic composition of microbial communities, a hypothesis supported by other studies [29, 30].

**Fig. 4:**
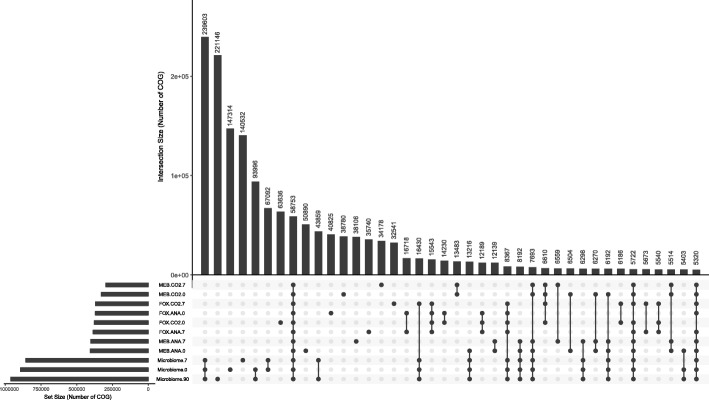
Distribution of 85% identity clusters of orthologous genes (COG) in culture-independent and culture-enriched microbiomes. Presence of COG in a given intersect is shown as a black dot in the intersection table at the bottom of the figure. The barplot at the top represents the number of COG in each intersection. The barplot on the left indicates the number of COG in each sample condition. The number at the end of conditions identifies the timepoint of sample collection (0, 7, or 90 days)

Nonetheless, the 1,581,710 putative genes stringently annotated as metabolic enzymes constitute only 7.3% of total genes detected in CIM and CEM. We thus generated clusters of orthologous genes (COG) using all 21,715,728 predicted genes. We used a clustering threshold of 85%, creating 2,009,496 COG. This approach allowed a comparison of the total gene content of microbial populations without function selection bias. The distribution of these COG between culture conditions and time points indicated a different distribution than that based on enzymes and pathways (Fig. 4). Indeed, while pathways and EC-annotated enzymes are in general present under all conditions, 48.9% of COG were specific to uncultured microbiomes and only 2.9% were observed in all four selective culture conditions and in the direct microbiomes. Also, 16.7% of COG were observed in a single CEM condition and time point. These results indicate that differences in microbiome gene content are for the most part not associated with metabolism-related genes, but with genes that have other functions. It explains in part why software that extrapolates metabolic pathways from 16S metabarcoding data perform appropriately for metabolism, but cannot assess the accessory genome and thus do not explain all phenotypes. Indeed, the health impact of gut microbes could be mediated by accessory genes such as those conferring antibiotic resistance.

### Mining assemblies for antibiotic resistance genes

We annotated a total of 13,218 putative resistance genes with at least 70% amino acid identity to genes known to confer resistance to antibiotics (Additional file 8: Table S4). These genes could be classified into 224 types. More than 98% of putative resistance genes were located on contigs for which taxonomic origin could be inferred (Additional file 9: Table S6 and Additional file 10: Table S7). A total of 3043 resistance genes were found near insertion sequences (Additional file 11: Table S5) and were assigned to 149 different types. Culture-enriched microbiomes allowed observation of 1.3 times more resistance genes than CIM.

Species with the highest number of resistance genes annotated when summing the assemblies of all tested conditions were *Escherichia coli* (5386 potential resistance genes on *E. coli* contigs), *Enterobacter cloacae* and related strain ST3 (2098), *Staphylococcus aureus* (297), *Bacteroides uniformis* (251), *Campylobacter jejuni* (229), *Klebsiella pneumoniae* (222), and *Enterococcus faecalis* (214). To evaluate the risk of these species harboring acquired antibiotic resistance genes in our antibiotic-exposed participants, we compared the antibiotic genes annotated on these contigs with the antibiotic resistance genes found in the pangenome of these species from publicly available datasets in NCBI Genomes (Table 1). Antibiotic resistance genes shared by more than 95% of the genomes of a species (the “soft-core” genes) were thus expected to be present in microbial communities that contain this species. Genes that are only found in a small subset of genomes, the flexible or accessory genome (5% to 95%) and the rare genes (< 5%), are more likely the product of HGT and were considered potentially acquired accessory genes in the following analysis [31]. However, it is not possible to use this approach to determine if this acquisition is recent or ancient.

**Table 1:** Comparison of antibiotics resistance genes from culture-independent and culture-enriched microbiomes to the pangenome of selected species

		*Bacteroides uniformis*	*Campylobacter jejuni*	*Enterobacter cloacae*	*Enterococcus faecalis*	*Enterococcus faecium*	*Escherichia coli*	*Staphylococcus aureus*
Reference genomes	Number of genomes of species for panresistome construction	14	458	432	246	119	3346	6632
	Types of resistance genes found in species (total resistome)	29	29	225	79	172	296	112
	Types of resistance genes present in > 95% genomes of species (core resistome)	0	2	34	0	0	35	12
	Types of resistance genes present in < 95% and > 5% genomes of species (accessory resistome)	9	5	35	21	21	23	18
	Types of resistance genes present in < 5% genomes of species (Rare resistome)	20	22	156	58	151	238	82
CIM and CEM	Number of CIM or CEM positive for resistance genes found in the context of species	157	174	39	124	30	146	101
	Types of resistance genes found in the context of the species in CIM or CEM (total resistome)	9	3	44	7	7	68	10
	Types of resistance genes found in the context of the species in CIM or CEM and present in > 95% genomes of species (core resistome)	0	0	35	0	0	35	0
	Types of resistance genes found in the context of the species in CIM or CEM and present in < 95% and > 5% genomes of species (accessory resistome)	6	1	6	5	5	22	4
	Types of resistance genes found in the context of the species in CIM or CEM and present in < 5% genomes of species (rare resistome)	0	0	3	2	1	10	4
	Types of resistance genes found in the context of the species in CIM or CEM and not found in reference genomes	3	2	0	0	1	1	2

**Fig. 5:**
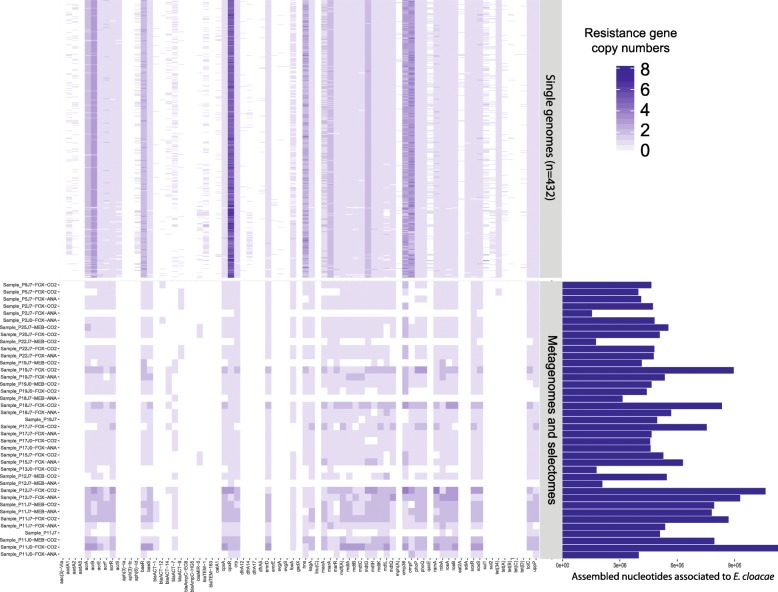
Comparison of resistance gene content associated with *Enterobacter cloacae* contigs and genes found in 432 *E. cloacae* whole genome sequences. The color of the heatmap indicates the copy number of each resistance gene. The upper part of the figure presents the distribution of resistance genes in whole genomes. The lower part of the figure shows the distribution of the same genes in contigs derived from CIM or CEM and identified as *E. cloacae.* The barplot on the right side shows the sum of the length of the contigs associated with *E. cloacae* in each CIM or CEM. The order of the genes is the same as in Fig. 6, meaning that the figure also includes *E. coli* resistance genes which may be absent from *E. cloacae* genomes, CIM, or CEM samples. The number after P identifies the participant (*n* = 24), and the number after J identifies the time point of sample collection (0 or 7 days). Only positive samples are shown in the figure

A total of 44 resistance gene types were found in contigs suspected of originating from *E. cloacae* based on k-mer content similarity*.* We investigated these genes using the pangenome of 432 publicly available sequences of *E. cloacae* genomes by annotating these genomes using the MERGEM database (Fig. 5, upper part). On average, *E. cloacae* genomes harbored 40 resistance genes. Thirty-four *E. cloacae* putative resistance genes found in CIM or CEM belonged to the core genome of this species, because they were found in more than 95% of the sequenced *E. cloacae* genomes (Fig. 5, lower part). Moreover, six of the non-core resistance genes found in our samples were the chromosomally encoded *bla*ACT beta-lactamase genes (also annotated as *bla*MIR), which represent different alleles of a gene that is present in all strains. These alleles are linked to whole genome phylogeny of *E. cloacae* (Additional file 12: Figure S5). The 3 remaining accessory resistance genes (*mdtL*, *fosA*, *ompF*) were prevalent in the pangenome (present in more 70% of samples). Therefore, we propose that the *E. cloacae* strains observed in the participants after cefprozil exposure did not carry clinically important transferable resistance genes. Still, core resistance genes could have been involved in the thriving of *E. cloacae* in the microbiomes after antibiotic treatment [9]. It is of note that a large proportion of the reference *E. cloacae* genomes were from clinical isolates that were obtained through screening for multidrug-resistant *Enterobacteriaceae* [32]. This could explain why the reference *E. cloacae* genomes contained more resistance genes than the strains observed in the microbiome of healthy individuals.

**Fig. 6:**
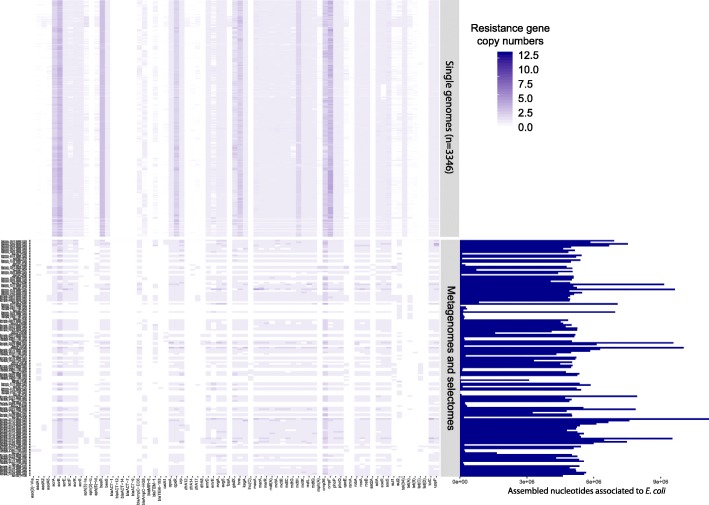
Comparison of resistance gene content associated with *Escherichia coli* contigs and genes found in 3346 *E. coli* whole genome sequences. The color of the heatmap indicates the copy number of each resistance gene. The upper part of the figure presents the distribution of resistance genes in whole genomes. The lower part of the figure shows the distribution of the same genes in contigs derived from CIM or CEM and identified as *E. coli.* The barplot on the right side shows the sum of the length of the contigs associated with *E. coli* in each CIM or CEM. The order of the genes is the same as in Fig. 5, meaning that the figure also includes *E. cloacae* resistance genes which may be absent from *E. coli* genomes, CIM, or CEM samples. The number after P identifies the participant (*n* = 24) and the number after J identifies the time point of sample collection (0, 7, or 90 days). Only positive samples are shown in the figure

We performed a similar analysis on contigs presumably originating from *E. coli* and found 68 families of resistance genes in putative *E. coli* contigs*.* On average, *E. coli* genomes harbored 46 resistance genes. We compared the putative resistance gene content of these contigs with the resistance genes found in 3346 *E.coli* genomes (Fig. 6). We also observed a large intrinsic resistome in *E.coli*, *as* 35 genes are shared by more than 95% of genomes. Two groups of *bla*AmpC genes were observed in the microbiomes and samples could be delineated based on alleles, which correlated with the population structure, a behavior similar to *bla*ACT in *E. cloacae* (Additional file 13: Figure S6). However, by contrast to *E. cloacae*, 33 types of putative resistance genes were found in only a subset of microbiomes and were present in a limited number of the *E. coli* genomes (< 95%). Moreover, accessory resistance genes and mobile elements are physically closer on chromosomes in *E. coli* than in *E. cloacae* (Additional file 14: Figure S7). These results suggest that those genes are part of the accessory *E. coli* genome and could have been acquired by horizontal gene transfer.

A total of 197 putative resistance genes were potentially located in *Enterococcus faecalis a*nd belonged to 7 resistance gene types. We thus investigated how the resistance genes observed in CIM or CEM were related to those observed in *E. faecalis* (246) and *E. faecium* (119) genomes available in public databases. No annotated resistance genes were part of the core genome for these two species. Respectively, 73% of *E. faecalis* and 88% of *E. faecium* resistance genes were found in less than 5% of sequenced genomes, suggesting that they could have been acquired by horizontal gene transfer. Five putative resistance genes found in the microbiome were part of the accessory genome of *E. faecalis*, and two were rare genes in this population. Similarly, in *E. faecium*, 5 of the 7 observed resistance genes were part of the accessory genome, 1 was rare, and 1 (aac(6’)-Ii) was not observed in *E. faecium* sequenced genomes.

We observed only accessory (< 95%) or rare (< 5%) resistance genes in *Staphylococcus aureus* genomic context in the CEM and CIM and no resistance genes were part of the core *S. aureus* resistome. Further investigation of putative *S. aureus* contigs using blastn on NCBI indicate potential similarity with taxa such as uncultured bacteria, *Enterobacter, Campylobacter*, or *Ruminococcus*, or sequences from plasmids*.* This is concordant with the low relative abundance of *Staphylococcus* (from a background signal to 0.3%) in the samples and suggests that the contigs identified as *S. aureus* could be wrongly annotated due to an incomplete reference database.

## Discussion

This study was designed to investigate how different, but complementary, stool culture conditions allowed the sequencing and analysis of bacteria that could not be properly sequenced using deep culture-independent microbiome sequencing. Using the MCDA broth as culture media, we have incubated stool samples in anaerobic and 5% CO_2_ conditions to determine how aerobic or anaerobic culture-enriched different bacteria. The two atmospheric conditions were also incubated in the presence or absence of the cefoxitin antibiotic to enrich for antibiotic-resistant bacteria. Overall, we found that sequencing broth culture of stool samples (CEM) allowed assembly of genomes that could not have been assembled using only culture-independent microbiome sequencing (CIM). For example, our CEM approach allowed assembly of the genome of *Enterobacteriaceae*, such as *E. coli, E. cloacae*, and *K. pneumoniae,* presenting abundances often lower than 1% in gut CIM. Indeed, *E. coli* had lower than 0.1% relative abundance in CIM, but was on average 15.2% of the community in MEB-CO_2_ and 6.2% in MEB-ANA. This was to be expected as *E. coli*, although not dominant in the gut microbiome, is well adapted for growth in vitro. The 5% CO_2_-enriched atmosphere allowed the growth of aerobes such as *Pseudomonas* and *Brevibacterium*. Moreover, there is a core of genomes that can be observed in CIM and CEM.

Notable gut microbiome bacteria like *Akkermansia* or *Prevotella* were not observed in CEM while they were prevalent in CIM. We suspect that the culture conditions used in this study were not appropriate for these species, as they are known to be difficult to culture. In addition, sample collection, storage, and preparation may have affected the taxa that were cultured in CEM and account for some differences between CIM and CEM. The extraction method for CIM and CEM was different as extraction from feces and bacterial culture have different requirements. However, bead-beating was used for cell lysis in both cases. Exposure to oxygen may have affected obligate anaerobic bacteria during sample collection and culture preparation. The genome of these bacteria could then have been present in the fecal samples, but the microorganism may not have been able to grow in culture [33]. The composition of culture media would also affect the community composition. For example, MCDA broth did not include short-chain fatty acids, which are required for the grown of some anaerobic bacteria like *Roseburia* [33]. In addition, some bacterial species may have faster growth rates in the selected culture conditions, which may favor them in the obtained CEM compared to bacteria with slower growth.

Nonetheless, broth culture under 5% CO_2_-enriched atmosphere, and anaerobic culture shared 57% of the genera found at more than 1% of the cultured community, many of these taxa being obligate anaerobes such as *Parabacteroides*, *Ruminococcus*, *Porphyromonas*, or *Bilophila* [28]. Although the medium does not contain oxygen reducing agents including those found in thioglycollate broth [34], oxygen reduction could be occurring. Indeed, through the growth of certain taxa in the community, oxygen at the bottom of the tube could be consumed or detoxified, thus allowing the growth of anaerobic bacteria. Since the 15-ml broth culture tubes were not shaken (nor stirred) and incubated for a period of 7 days, a diminishing O_2_ gradient might have been established, therefore explaining the apparent growth of anaerobes in CO_2_. In addition, bacteria thought to be strictly anaerobic could adapt to microaerobic conditions by modulating gene expression, as shown for *Porphyromonas gingivalis* [35].

The broth culture conditions used in this study permitted the growth of microbial communities that could include previously difficult to sequence taxa due to their low relative abundance in the microbiome. For example, the MEB-CO_2_ condition allows assembling the genomes of several genera that have been associated with the mucus layer of the intestine [28] or bacteria known to reside in the small intestine or in the interfold regions [36]. We thus suggest that the selection of culture media based on bacterial taxa of interest could allow for a deeper insight into specific subcommunities of the gut microbiome. Moreover, the sequencing depth of CEM (3.2 Gb/CEM) was on average fourfold lower than CIM (12.6 Gb/CIM), thus four times less expensive than CIM to recover additional genomes. The use of carefully chosen selective media could prove a viable method for the metagenomic characterization of specific portions of the microbiota without invasive procedures and with reduced sequencing effort. This approach is complementary to culture-independent microbiome sequencing and can be performed after initial metagenomic profiling. In addition to recovering the genome of lower abundance bacteria, recovery of specific taxa in CEM would suggest that they were viable in the stool sample, a notion that cannot be inferred from CIM [37]. A major limitation of this growth-based approach is that culture limits our capacity to do a quantitative comparison of the relative abundance of bacteria between CEM. However, CIM or 16S metataxonomics would still retain this information and could be used as a reference.

Culture-enriched microbiome analysis allowed the evaluation of the gene content of specific subcommunities of the gut microbiome, as the assembly of CIM and CEM permitted the characterization of the functional profile of bacteria. We annotated metabolic pathway enzymes on the assembled contigs to determine which pathways were detected in CIM or CEM. Then, we identified the putative taxonomic origin of contigs to infer to which bacterial functions they were most probably associated. Contigs can be properly identified based on adequate representation of the gut microbiome in the reference genome database used for taxonomic profiling [25]. It remains possible that this identification could be biased by database composition or by horizontal gene transfer. We first observed that the four culture conditions and CIM sequencing shared a large pool of similar enzymes, which are associated with diverse microbial origins and functions. Some enzymes were associated with specific culture conditions and, for the most part, with specific taxa. This is in accordance with findings that show an association between the functions found in the microbiome and taxonomical distribution [29, 30]. However, other work indicates that mobile genes could often be structured according to ecological context rather than by taxonomy [38, 39], which is coherent with the overall distribution of genes, regardless of functions, between samples and conditions.

This last paradox is especially true for antibiotic resistance genes, which can both be intrinsic to the core genome of a species or acquired through horizontal gene transfer [40]. Studies of antibiotic resistance genes in the microbiome often consist in catalogs of genes, thus overlooking the difference in risk associated with genes prone to HGT compared to genes intrinsic to the core genome [41]. For example, in our previous work, we observed several antibiotic resistance genes associated with *E. cloacae* genomes reconstituted from metagenomic sequencing after exposure of volunteers to the antibiotic cefprozil [9]. However, quantifying the HGT potential of these genes was problematic, as short-read sequencing does not provide an easy way to determine whether genes are intrinsic or have been acquired by horizontal transfer. As the CEM approach allows assembly of a large array of genomes from *Enterobacteriaceae*, we developed a method to infer if antibiotic resistance genes were intrinsic or the result of potential HGT. First, we generated the panresistome of a species by annotating a collection of genomes obtained from public databases. Then, we compared the gene content of contigs identified as originating from this species with the panresistome of the species. This approach allowed identification of genes belonging to the core genome of a species, thus suggesting that they have a low probability of being mobile. After the analysis of the *E. cloacae* panresistome, we found that resistance genes from this species were associated with its core genome and not with potentially mobile elements. By contrast, the *E. coli-*related contigs contained numerous resistance genes belonging to the accessory genome, some of which were rare in the *E. coli* species population, suggesting that some *E. coli* resistance genes in the microbiome of our study participants could be mobile, or at least strain-specific. Genes belonging to the core genome of a species are often associated with essential functions. As antibiotics target core functions of the bacteria, many resistance genes can be part of the core genome of some species. For example, the *blaACT* of *E. cloacae* or *blaAmpC* of *E. coli* are part of the peptidoglycan biosynthesis machinery in *Enterobacteriaceae*, thus conferring upon these bacteria intrinsic resistance to some antibiotics [42]. In contrast, accessory resistance genes are optional and not essential for the survival of a species in normal conditions. These can however be selected in the presence of a selective agent such as an antibiotic.

The pangenome method can also diagnose the assembly of a species, as the coherence between the core genes found in reference genomes and those found in metagenomes indicate that this species has an appropriate sequencing coverage. As seen with *S. aureus,* discordances could suggest either an incorrect annotation of contigs or an inability to assemble the complete genome of a species. Still, the CEM method has some drawbacks such as inability to take into account resistance genes in contigs that were not associated with a given species because of low coverage, absence of representative strains in reference databases or presence of genes in contigs associated with other species because of HGT. Moreover, the origin of the reference genomes should be taken into account during the interpretation of pangenome-derived analysis. Clinical isolates from a species can harbor a different resistome than commensals. For instance, a large proportion of the *Enterobacter cloacae* genomes used as a reference in this work came from a study screening for multidrug-resistant *Enterobacteriaceae* [32]. As such, this *E. cloacae* genome collection may not be fully representative of the diversity of this species. In contrast, the microbiomes included in this study are from healthy individuals without underlying conditions; therefore, the *E. cloacae* strains observed in CIM and CEM may not be pathogenic. Still, this approach allowed to determine if changes in antibiotic resistance genes in microbiomes were linked to taxonomy. Comparison to the pangenome of species is a tool that can be used to mitigate the risk of overinterpreting results by putting them in a broader context than in a single study. Further work in microbiome strain epidemiology will improve the interpretation of microbiome data.

Other approaches for the detection of antibiotic resistance genes from microbiome samples are available and provide complementary information on the resistome. For example, quantitative PCR allows detection and quantification of the abundance of specific resistance genes, but without determining their sequence or genetic context [33]. Recently, Lanza and collaborators showed that specific sequence capture using a large collection of antibiotic resistance gene probes was a sensitive method to identify the resistome of fecal samples and to determine its genomic sequence [43]. One drawback of this approach is that it does not provide the genomic context and taxonomic identification of the origin of these genes. For improved genome context, read-cloud sequencing combined to CIM or CEM methodologies could provide contiguous assemblies of low-abundance genomes [44].

## Conclusion

In this work, we addressed two important issues of microbiome analysis. First, we suggested a strategy to investigate the genome of bacteria of low relative abundance in the gut microbiome without overly deep sequencing. The culture conditions we used for CEM were especially efficient in recovery of *Enterobacteriaceae.* The use of other liquid culture media could contribute to an improved genotyping of less-abundant gut bacteria, for example, those associated with the mucosa. Second, we interpreted the potential importance of resistance genes from the gut microbiome by exploiting existing genomic information from public databases. As more bacterial genomes become available, it is essential to consider the information they provide in interpreting results from metagenomic studies, not limited to resistance gene analysis. Defining if a gene is part of the core genome of a species is an approach that can help interpret genomes recovered from microbiomes. This big data approach improves the information that can be extracted from microbial population studies.

## Additional files

Additional file 1: **Table S1.** Sequencing statistics of culture-enriched microbiomes. (XLSX 26 kb)

Additional file 2: **Figure S1.** Diversity and abundance distribution of bacterial families in culture-independent or culture-enriched microbiomes. A) Beanplots of the Shannon diversity of CIM or CEM samples profiled at the rank of species. B) Abundance distribution of bacterial families in CIM or CEM. For each curve, the families are ordered from the most abundant to the least abundant (for the 25 first taxa). Data shown are the average proportion of each taxon for each condition. (PDF 432 kb)

Additional file 3: **Table S2.** Taxonomic profiling at the rank of species. (XLS 6107 kb)

Additional file 4: **Table S3.** Assembled genome size per species. (XLSX 377 kb)

Additional file 5: **Figure S2.** Relationship between the depth of sequencing and sum of the assembled contigs associated with a species for 225 species. Each panel represents a different species. The *y* axis represents the nucleotides assembled into contigs associated with a species. The *x* axis is the estimated number of nucleotide sequences for the species (proportion of the species in the microbiome multiplied by the number of nucleotides sequenced for this sample). The horizontal lines indicate 1 million nucleotides. As represented in the legend, the color of the points relate to the CIM or CEM conditions from which each point originates. (PDF 3014 kb)

Additional file 6: **Figure S3.** Distribution of pathways in culture-independent or culture-enriched microbiomes. Pathways are considered present in a condition if at least one EC number of this pathway is present in at least one sample of said condition. (PDF 136 kb)

Additional file 7: **Figure S4.** Proportion of samples and enzymes positive per cluster per condition. For each combination of enzyme clusters and experimental conditions, the proportion of positive enzymes compared to the total size of the combination was calculated. A value of 1 indicates that all samples were positive for all enzymes. A value of 0 indicates that no sample in a given condition was positive for any enzyme from the cluster. (PDF 305 kb)

Additional file 8: **Table S4.** Number of resistance genes observed per culture-independent or culture-enriched microbiomes with 70 percent identity to reference resistance genes from MERGEM. (XLSX 14 kb)

Additional file 9: **Table S6.** Potential species origin of antibiotic resistance genes. (XLSX 25 kb)

Additional file 10: **Table S7.** Families of antibiotic resistance genes associated with potential species of origin. (XLSX 154 kb)

Additional file 11: **Table S5.** Number of resistance genes observed per microbiome and selectome with 70 percent identity to reference resistance genes from MERGEM. (XLSX 14 kb)

Additional file 12: **Figure S5.** Association between genome content clustering and content in resistance genes for 432 *Enterobacter cloacae* whole genomes. The color of the heatmap indicates the copy number of each resistance gene in whole genomes. Hierarchical clustering of the genomes is based on the comparison of their content in k-mers using the Ray Surveyor software. (PDF 1449 kb)

Additional file 13: **Figure S6.** Association between genome content clustering and content in resistance genes for 3346 *Escherichia coli* whole genomes. The color of the heatmap indicates the copy number of each resistance gene in whole genomes. Hierarchical clustering of the genomes is based on the comparison of their content in k-mers using the Ray Surveyor software. (PDF 1465 kb)

Additional file 14: **Figure S7.** Distance in nucleotides between the core and accessory resistance genes from mobile elements in *E. coli* and *E. cloacae*. (PDF 175 kb)
