# Supplementary figures and images for: Culture-enriched human gut microbiomes reveal core and accessory resistance genes
Source: Microbiome. 2019 Apr 5;7:56. doi: 10.1186/s40168-019-0669-7 (PMC6451232; doi:10.1186/s40168-019-0669-7)

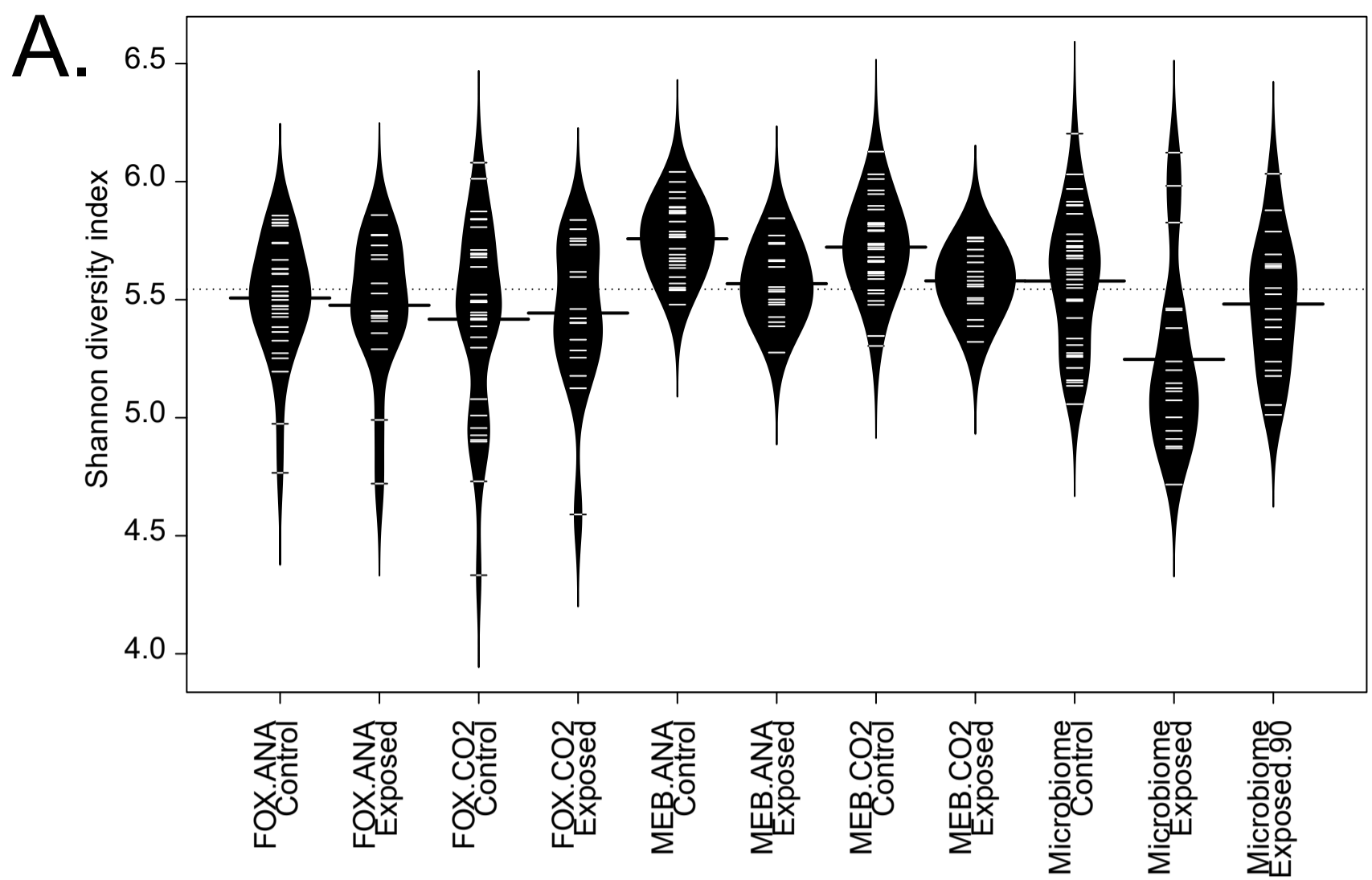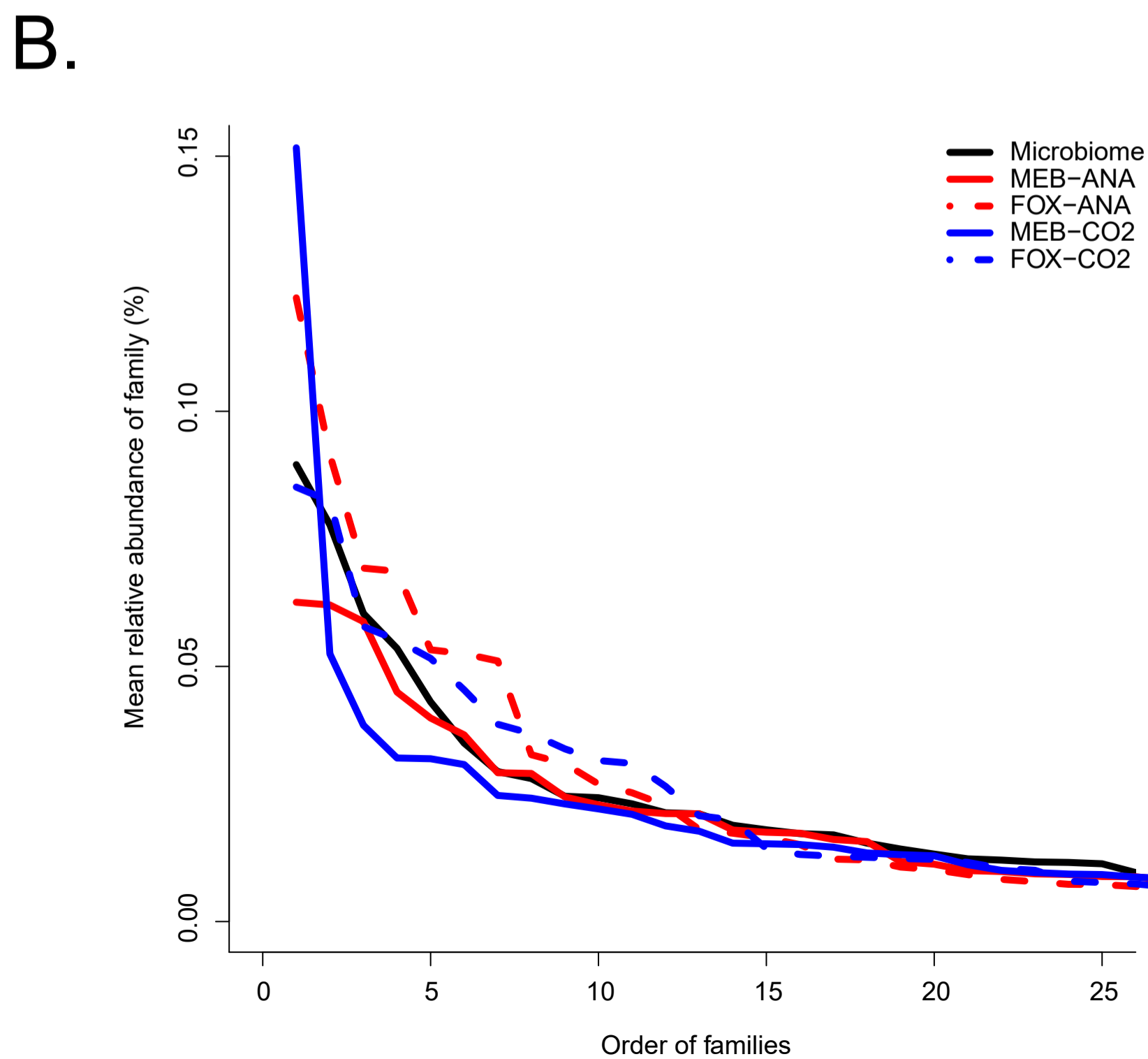

Supplement: Supplementary file 2 — Figure S1. Diversity and abundance distribution of bacterial families in culture-independent or culture-enriched microbiomes. A) Beanplots of the Shannon diversity of CIM or CEM samples profiled at the rank of species. B) Abundance distribution of bacterial families in CIM or CEM. For each curve, the families are ordered from the most abundant to the least abundant (for the 25 first taxa). Data shown are the average proportion of each taxon for each condition. (PDF 432 kb) [file 40168_2019_669_MOESM2_ESM.pdf]

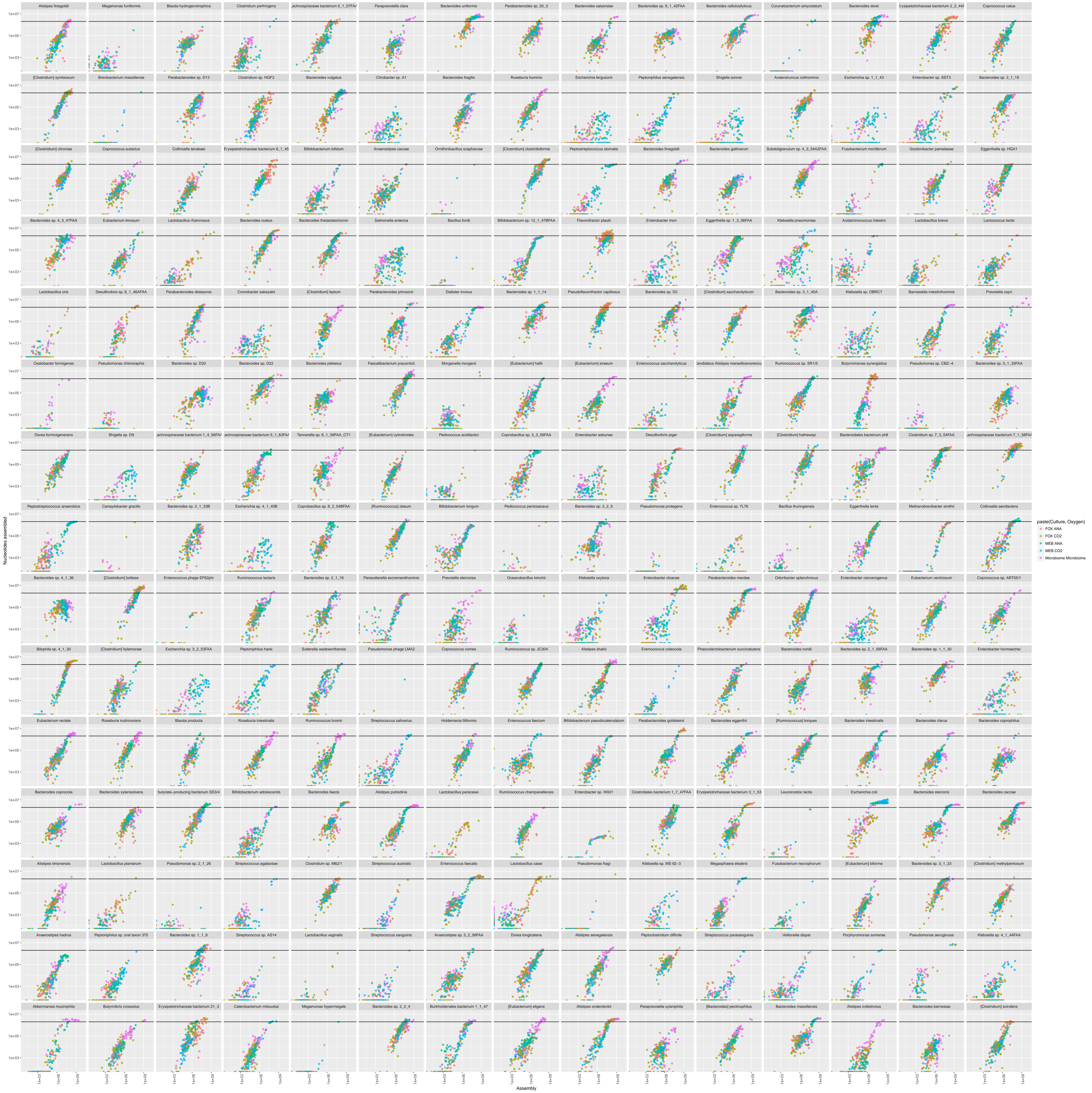

Supplement: Supplementary file 5 — Figure S2. Relationship between the depth of sequencing and sum of the assembled contigs associated with a species for 225 species. Each panel represents a different species. The y axis represents the nucleotides assembled into contigs associated with a species. The x axis is the estimated number of nucleotide sequences for the species (proportion of the species in the microbiome multiplied by the number of nucleotides sequenced for this sample). The horizontal lines indicate 1 million nucleotides. As represented in the legend, the color of the points relate to the CIM or CEM conditions from which each point originates. (PDF 3014 kb) [file 40168_2019_669_MOESM5_ESM.pdf]

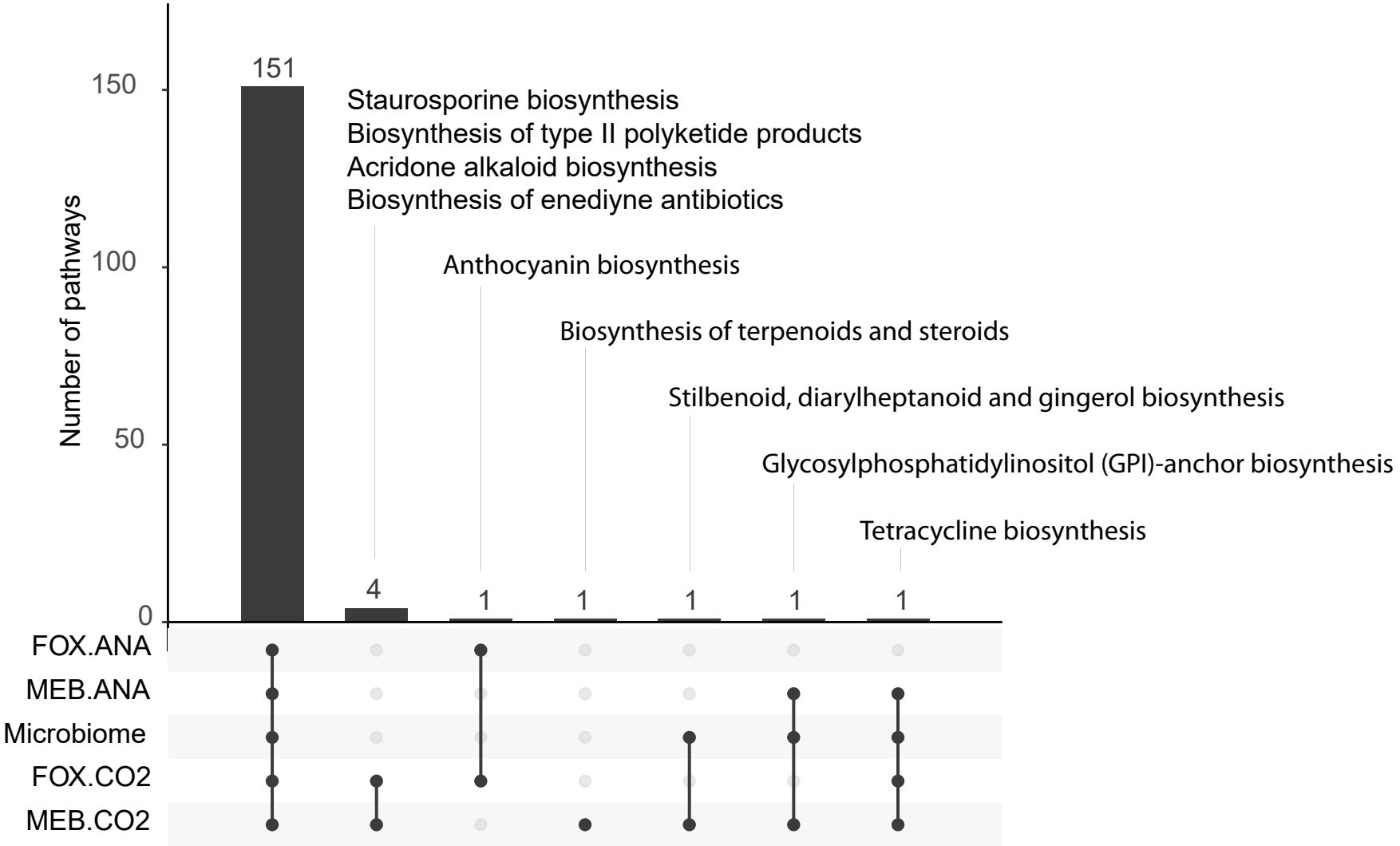

Supplement: Supplementary file 6 — Figure S3. Distribution of pathways in culture-independent or culture-enriched microbiomes. Pathways are considered present in a condition if at least one EC number of this pathway is present in at least one sample of said condition. (PDF 136 kb) [file 40168_2019_669_MOESM6_ESM.pdf]

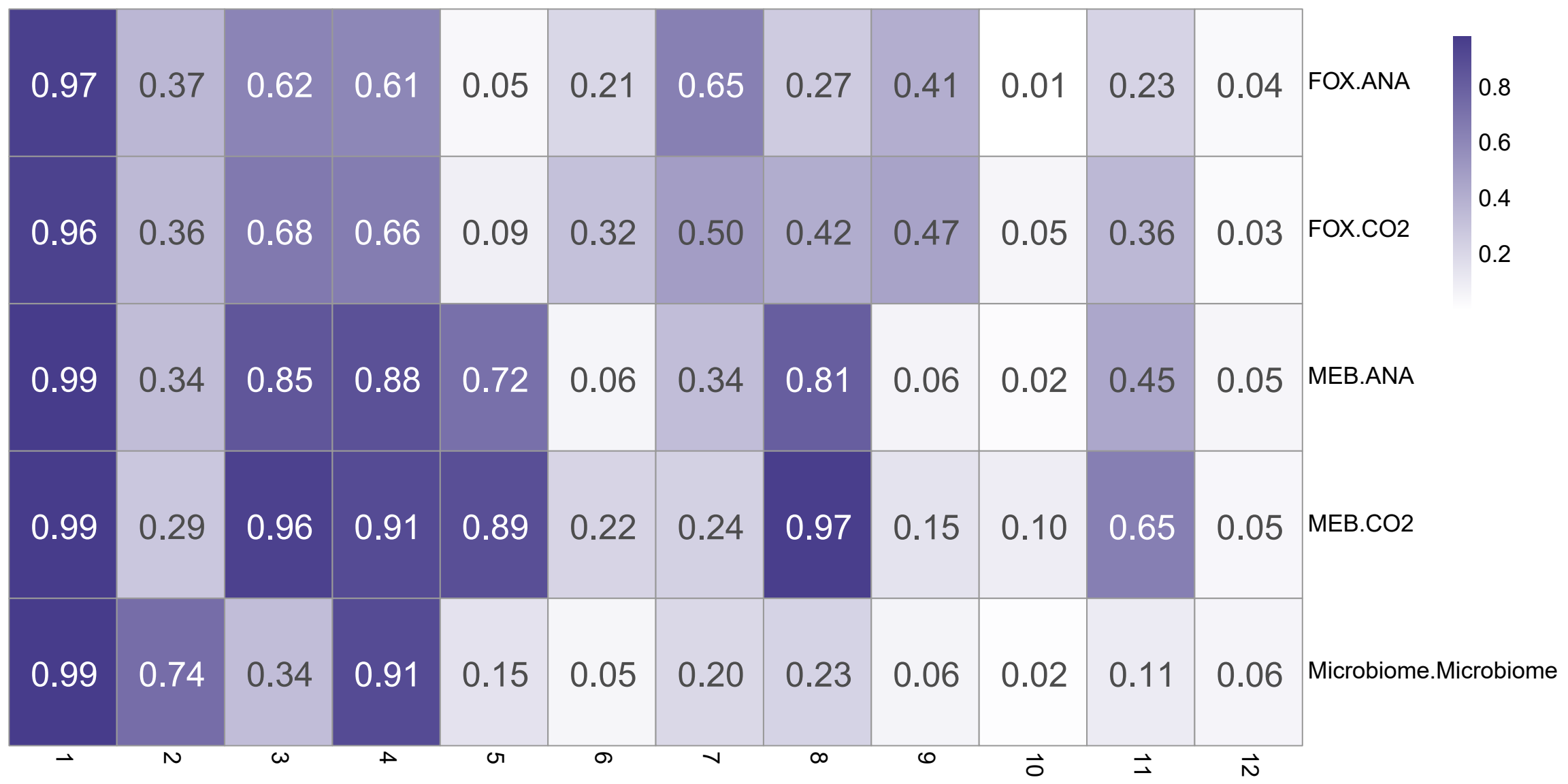

Supplement: Supplementary file 7 — Figure S4. Proportion of samples and enzymes positive per cluster per condition. For each combination of enzyme clusters and experimental conditions, the proportion of positive enzymes compared to the total size of the combination was calculated. A value of 1 indicates that all samples were positive for all enzymes. A value of 0 indicates that no sample in a given condition was positive for any enzyme from the cluster. (PDF 305 kb) [file 40168_2019_669_MOESM7_ESM.pdf]

Hierarchical clustering of the genomes  
based on complete k-mer content

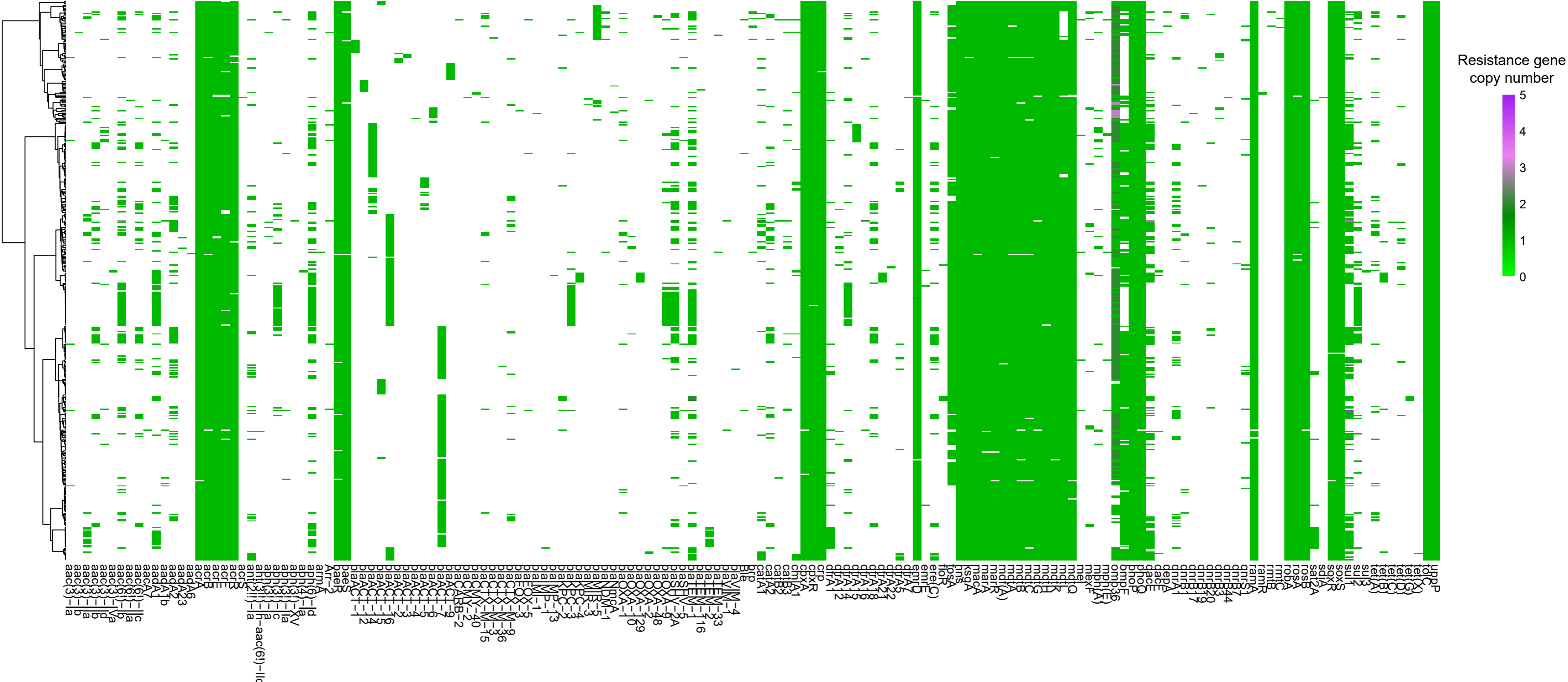

Supplement: Supplementary file 12 — Figure S5. Association between genome content clustering and content in resistance genes for 432 Enterobacter cloacae whole genomes. The color of the heatmap indicates the copy number of each resistance gene in whole genomes. Hierarchical clustering of the genomes is based on the comparison of their content in k-mers using the Ray Surveyor software. (PDF 1449 kb) [file 40168_2019_669_MOESM12_ESM.pdf]

based on complete k-mer content

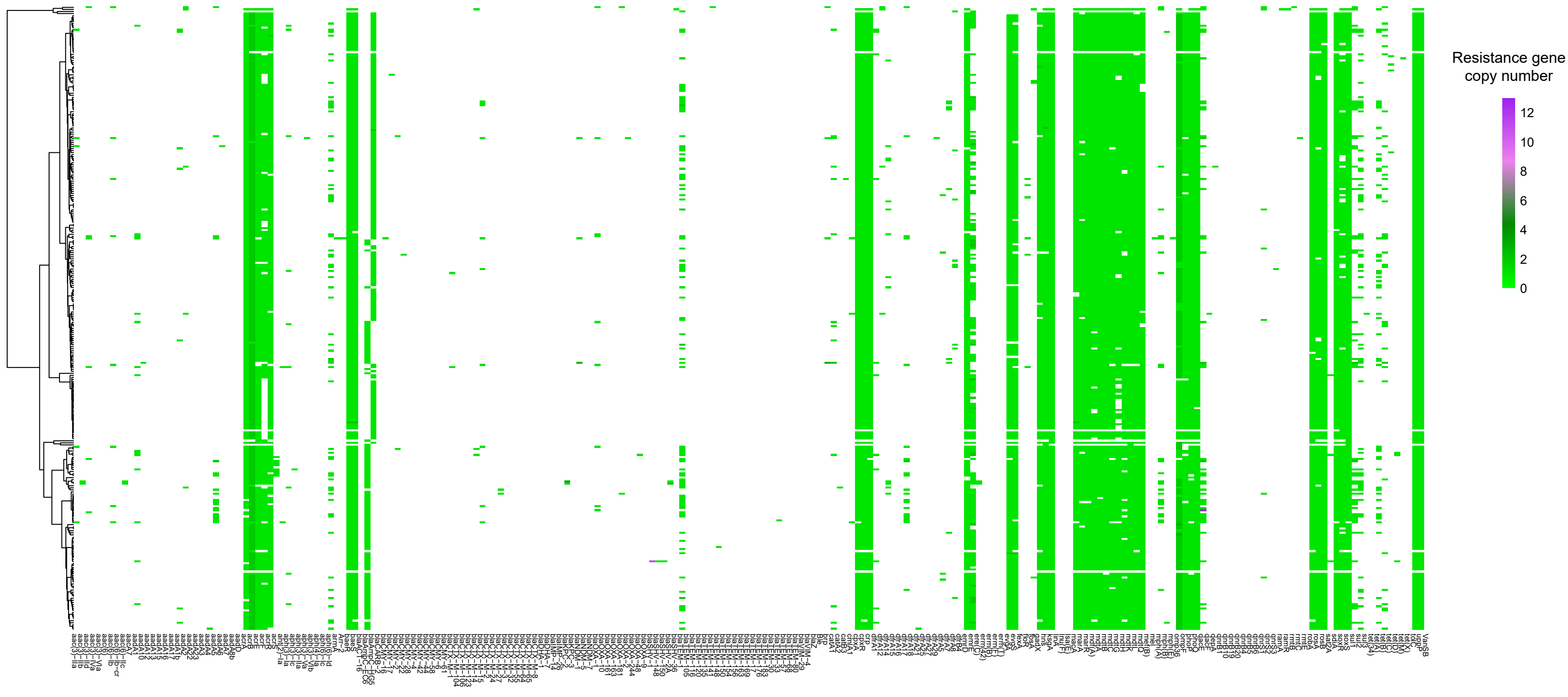

Supplement: Supplementary file 13 — Figure S6. Association between genome content clustering and content in resistance genes for 3346 Escherichia coli whole genomes. The color of the heatmap indicates the copy number of each resistance gene in whole genomes. Hierarchical clustering of the genomes is based on the comparison of their content in k-mers using the Ray Surveyor software. (PDF 1465 kb) [file 40168_2019_669_MOESM13_ESM.pdf]

Distance (nucleotides) between core and accessory resistance genes from mobile elements

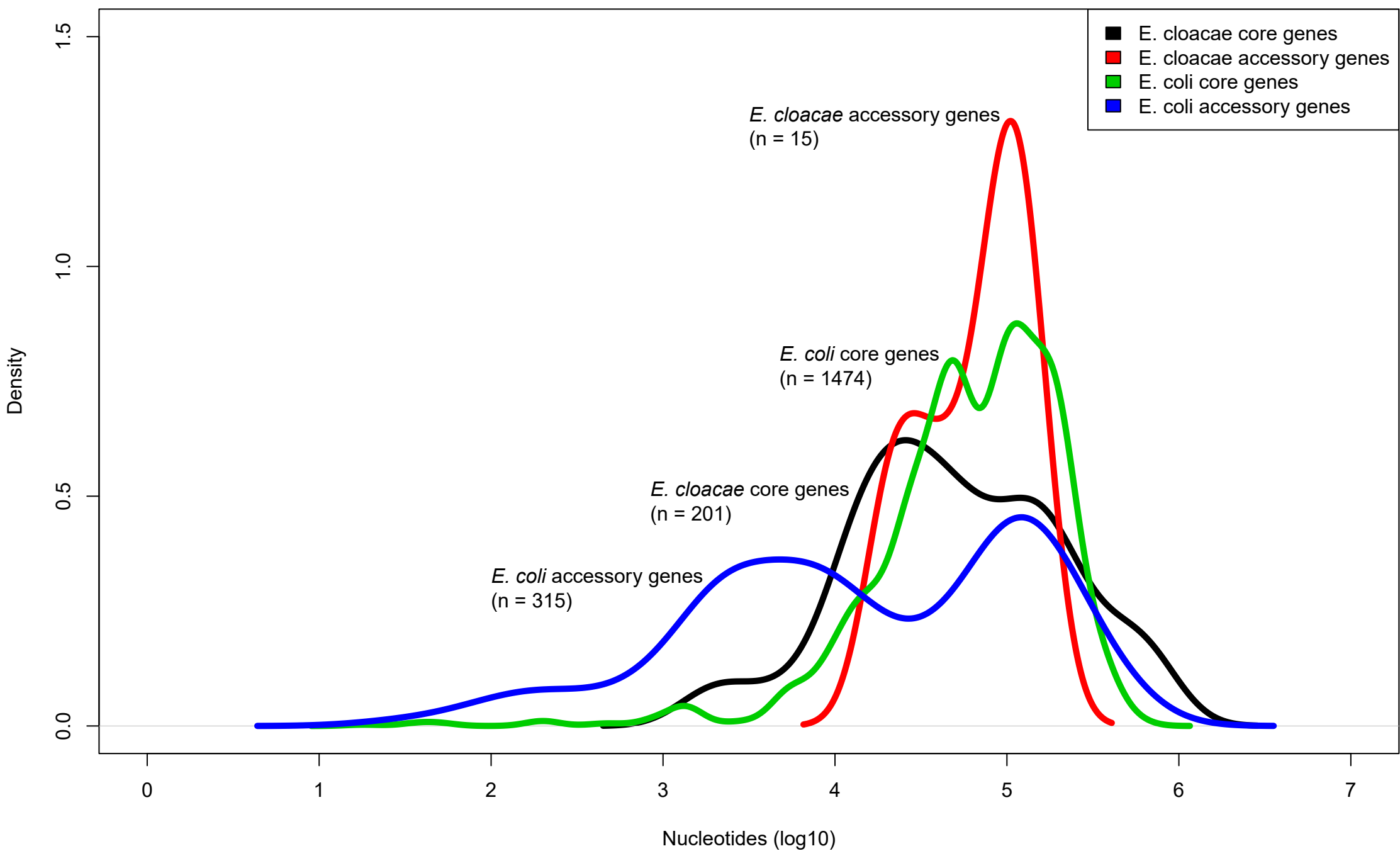

Supplement: Supplementary file 14 — Figure S7. Distance in nucleotides between the core and accessory resistance genes from mobile elements in E. coli and E. cloacae. (PDF 175 kb) [file 40168_2019_669_MOESM14_ESM.pdf]
